# Potential Therapeutic Strategies for Skeletal Muscle Atrophy

**DOI:** 10.3390/antiox12010044

**Published:** 2022-12-26

**Authors:** Li Huang, Ming Li, Chunyan Deng, Jiayi Qiu, Kexin Wang, Mengyuan Chang, Songlin Zhou, Yun Gu, Yuntian Shen, Wei Wang, Ziwei Huang, Hualin Sun

**Affiliations:** 1 Key Laboratory of Neuroregeneration of Jiangsu and Ministry of Education, Co-Innovation Center of Neuroregeneration, Medical College of Nantong University, NMPA Key Laboratory for Research and Evaluation of Tissue Engineering Technology Products, Jiangsu Clinical Medicine Center of Tissue Engineering and Nerve Injury Repair, Nantong University, Nantong 226001, China; 2 Department of Laboratory Medicine, Binhai County People’s Hospital, Yancheng 224500, China; 3 Department of Clinical Medicine, Medical College, Nantong University, Nantong 226001, China; 4 Department of Pathology, Affiliated Hospital of Nantong University, Nantong 226001, China; 5 Division of Sports Medicine and Adult Reconstructive Surgery, Department of Orthopedic Surgery, Nanjing Drum Tower Hospital, The Affiliated Hospital of Nanjing University Medical School, Nanjing 210008, China; 6 Department of Orthopaedic Surgery, Massachusetts General Hospital, Harvard Medical School, Boston, MA 02115, USA

**Keywords:** skeletal muscle atrophy, drug treatment, gene therapy, stem cell therapy, cytokine therapy

## Abstract

The maintenance of muscle homeostasis is vital for life and health. Skeletal muscle atrophy not only seriously reduces people’s quality of life and increases morbidity and mortality, but also causes a huge socioeconomic burden. To date, no effective treatment has been developed for skeletal muscle atrophy owing to an incomplete understanding of its molecular mechanisms. Exercise therapy is the most effective treatment for skeletal muscle atrophy. Unfortunately, it is not suitable for all patients, such as fractured patients and bedridden patients with nerve damage. Therefore, understanding the molecular mechanism of skeletal muscle atrophy is crucial for developing new therapies for skeletal muscle atrophy. In this review, PubMed was systematically screened for articles that appeared in the past 5 years about potential therapeutic strategies for skeletal muscle atrophy. Herein, we summarize the roles of inflammation, oxidative stress, ubiquitin-proteasome system, autophagic-lysosomal pathway, caspases, and calpains in skeletal muscle atrophy and systematically expound the potential drug targets and therapeutic progress against skeletal muscle atrophy. This review focuses on current treatments and strategies for skeletal muscle atrophy, including drug treatment (active substances of traditional Chinese medicine, chemical drugs, antioxidants, enzyme and enzyme inhibitors, hormone drugs, etc.), gene therapy, stem cell and exosome therapy (muscle-derived stem cells, non-myogenic stem cells, and exosomes), cytokine therapy, physical therapy (electroacupuncture, electrical stimulation, optogenetic technology, heat therapy, and low-level laser therapy), nutrition support (protein, essential amino acids, creatine, β-hydroxy-β-methylbutyrate, and vitamin D), and other therapies (biomaterial adjuvant therapy, intestinal microbial regulation, and oxygen supplementation). Considering many treatments have been developed for skeletal muscle atrophy, we propose a combination of proper treatments for individual needs, which may yield better treatment outcomes.

## 1. Introduction

Skeletal muscle is one of the largest, most dynamic, and plastic tissues in the body, comprising approximately 40% of the total body mass and containing 50–75% of overall body protein stores. Skeletal muscle is primarily responsible for body movement, protein storage, thermogenesis, metabolism, and visceral protection [1]. Mechanically, the major function of skeletal muscle is to convert chemical energy into mechanical energy to generate force and momentum, maintain posture, and produce movements that affect physical activities. Metabolically, skeletal muscle promotes basal energy metabolism, stores important substrates such as amino acids and carbohydrates, and generates heat to maintain core temperature. Skeletal muscle is also a library of amino acids required for the synthesis of organ-specific proteins by other organs, such as the skin, brain, and heart. Amino acids released by skeletal muscle are beneficial for maintaining glucose homeostasis under starvation conditions [2]. Given the above, the maintenance of skeletal muscle homeostasis plays a crucial role in maintaining good life and health. Various factors can disturb skeletal muscle homeostasis and thus trigger skeletal muscle atrophy, including diseases (such as diabetes mellitus, cancer, and chronic obstructive pulmonary disease), weightlessness, denervated disuse states, fasting, and aging [3,4]. Ageing is accompanied by a progressive decline in skeletal muscle mass and strength, which may lead to primary sarcopenia [5]. Skeletal muscle atrophy impairs the body’s ability to respond to stress and chronic diseases, severely reduces people’s quality of life, increases morbidity and mortality, brings a huge socioeconomic burden, and impacts patient prognosis [6]. Therefore, the prevention and treatment of skeletal muscle atrophy aroused great concern of scholars and clinicians. However, because the molecular mechanism of skeletal muscle atrophy is not fully understood, there is currently no effective treatment for skeletal muscle atrophy.

## 2. Methods

**Figure 1:**
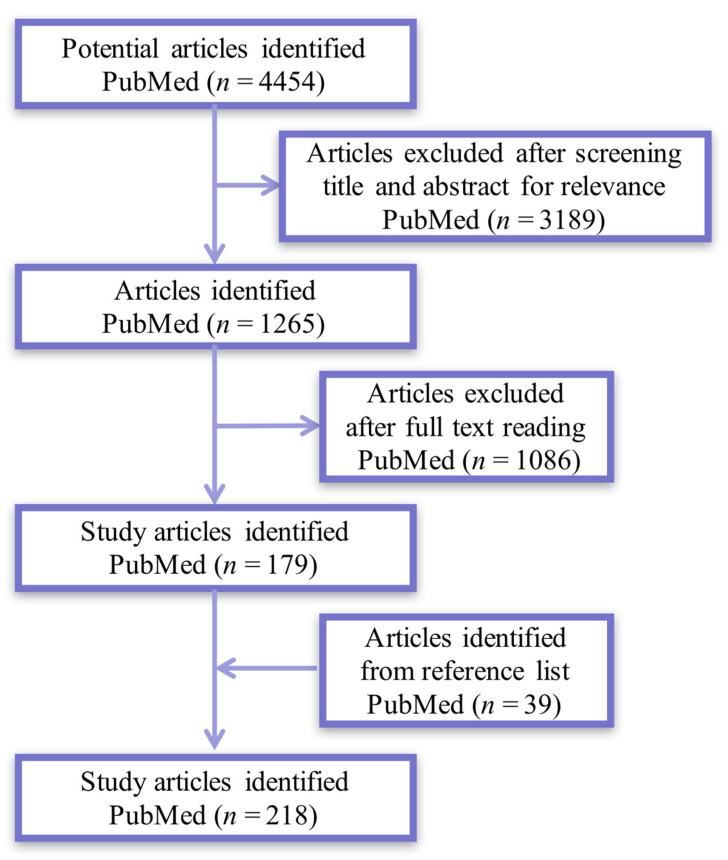
Flowchart of article screening.

The review article is based on a selective literature search in PubMed for publications of the five years from 2017 to 2022. The following keywords were used: “muscle atrophy” and “therapy”. Further targeted searches were performed including “Drug treatment”, “Gene therapy”, “Stem cell”, “physical therapy”, “cytokines”, and “Nutrition” as a therapy for muscle atrophy. The screening process was independently performed by two reviewers who were blind to each other’s results. First, we screened the articles based on the title and abstract. Then, we thoroughly read the full texts and screened. If there were disagreements on the inclusion of an article, a consensus was reached through the participation of the third reviewer. From the search results, we screened the reference lists of above included articles and added corresponding studies. After investigations of all searched articles, 179 manuscripts were finally selected to accomplish this review (Figure 1).

## 3. Molecular Mechanisms of Skeletal Muscle Atrophy

**Figure 2:**
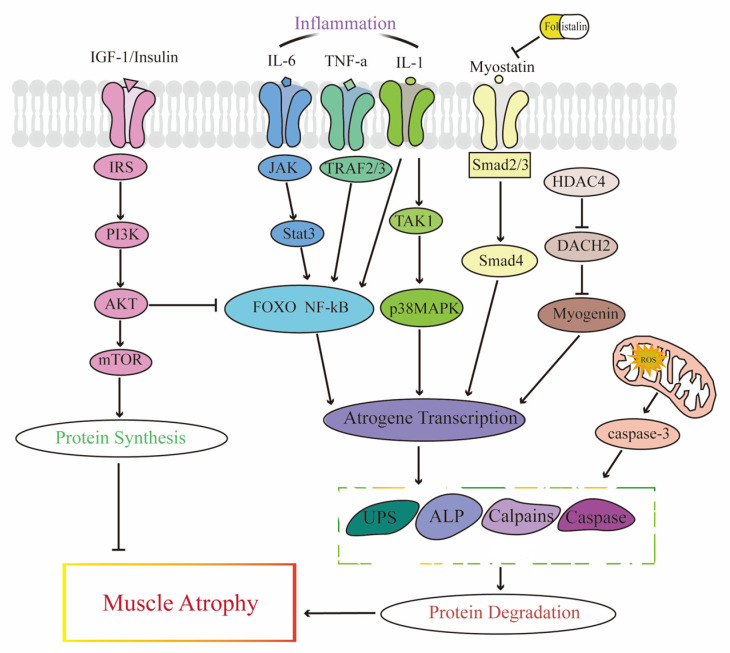
The molecular mechanism of skeletal muscle atrophy. ALP, autophagy–lysosome pathway; DACH2, dachshund homolog 2; FOXO, forkhead transcription factor O-box subfamily; HDAC4, histone deacetylase 4; IGF-1, insulin-like growth factor 1; IL-6, interleukin 6; IL-1, interleukin 1;IRS, insulin receptor substrate; IL, interleukin; JAK, janus kinase; mTOR, mammalian target of rapamycin; NF-kB, nuclear factor-kB; PI3K, phosphoinositide 3-kinases; ROS, reactive oxygen species; STAT3, signal transducer and activator of transcription 3; TAK1, transforming growth factor b-activated kinase 1; TNF-α, tumor necrosis factor-a; TRAF, TNF receptor-associated factor; UPS, ubiquitin–proteasome system.

The molecular mechanism of skeletal muscle atrophy is complex and has not yet been fully elucidated. The continuous renewal of muscle proteins is the result of the balance between protein synthesis and degradation in muscle tissue [7]. Decreased protein synthesis and increased proteolysis will trigger skeletal muscle atrophy (Figure 1). Increased oxidative stress, inflammation, and decreased mitochondrial function are considered important triggering signals for skeletal muscle atrophy caused by various diseases. This is because these phenomena can activate downstream proteolysis and inhibit protein synthesis, thereby causing skeletal muscle atrophy (Figure 2) [8,9]. Our previous study proposed for the first time that denervation-induced muscle atrophy can be divided into four stages: oxidative stress, inflammation, atrophy, and atrophic fibrosis. This can be explained as the loss of contractile function of the target muscle due to denervation, resulting in reduced blood perfusion and making the target muscle in a state of relative hypoxia. Then, excessive reactive oxygen species can generate and cause oxidative stress injury, which induces the production of a large number of inflammatory cytokines, such us IL-1, TNF-α, and IL-6. These inflammatory cytokines further activate inflammatory response pathways, including nuclear factor NF-κB, and JAK/STAT and TAK1/p38MAPK pathways initiate the expression of genes related to the downstream proteolysis system and cause atrophy of the target muscle [10]. Inflammation, oxidative stress and some other factors may increase Myostatin (MSTN) synthesis while excessive MSTN can activate Smad2/3 pathway, which subsequently promotes proteolysis, ultimately causing muscle atrophy [11]. There are four main pathways available for proteolysis, including ubiquitin-proteasome system (UPS), autophagic-lysosomal pathway (ALP), caspases, and calpains [12,13,14,15]. Complete protein degradation requires the synergy of these hydrolysis systems [16]. Caspase-3 and calpain mainly function to cleave target proteins into fragments while ALP and UPS further degrade proteins and protein fragments into amino acids [14]. UPS, ALP, and caspase-3 act as “erasers” to degrade and eliminate substrate proteins. In contrast, calpain can also hydrolyze its substrates at a limited number of protein sites to change or modulate its substrate structure and activity, enabling the substrate to further exert its biological functions [17]. IGF-1/Akt is the major pathway for protein synthesis in skeletal muscle. IGF-1 increases protein synthesis in skeletal muscle via the PI3K/Akt/mTOR pathways. PI3K/Akt can also inhibit the transcription of E3 ubiquitinligase by inhibiting FoxOs, thereby slowing proteolysis [18]. Histone deacetylase 4 (HDAC4) is a negative regulator of Dach2, which subsequently regulated the expression of MYOG. HDAC4 Knockdown can alleviate denervation-induced muscle atrophy by inhibiting myogenin-dependent atrogene activation [19]. Overall, the molecular mechanism of muscle atrophy is very complex and has not been fully elucidated so far. Further studies are still needed to provide potential targets and new therapeutic strategies for muscle atrophy.

## 4. Treatments

**Figure 3:**
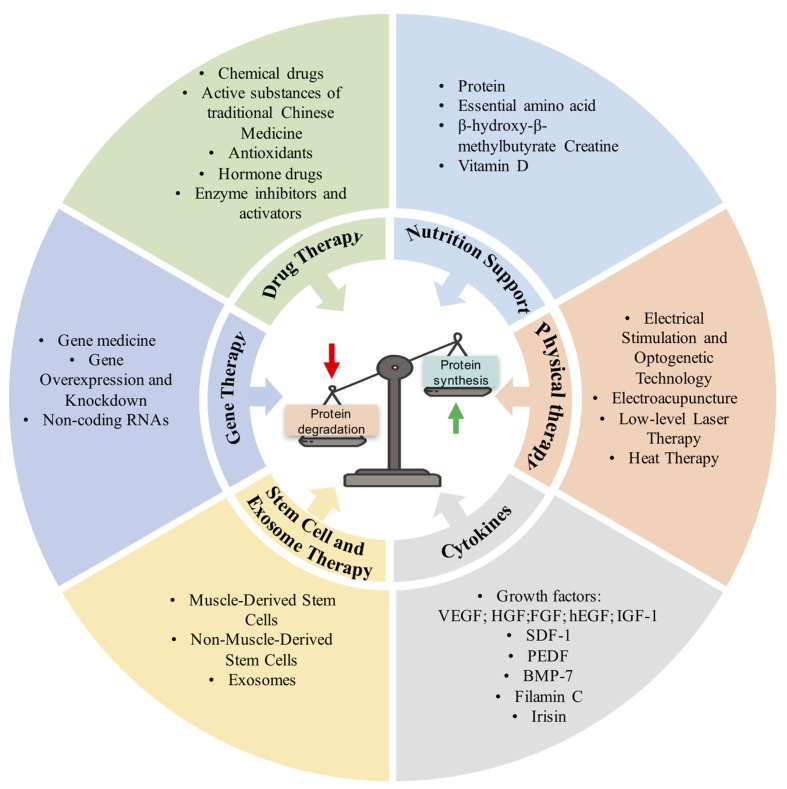
The current potential therapeutic strategy for skeletal muscle atrophy. VEGF, vascular endothelial growth factor; HGF, hepatocyte growth factor; FGF, fibroblast growth factor; hEGF, human epidermal growth factor; IGF-1, insulin-like growth factor 1; SDF-1, stromal cell-derived factor-1; PEDF, pigment epithelium-derived factor; BMP, bone morphogenetic protein-7.

Skeletal muscle atrophy is characterized by a marked reduction in muscle mass, a reduced quality of life for patients, and increased morbidity and mortality. Muscle atrophy is mainly caused by the imbalance of protein synthesis and degradation. Oxidative stress and inflammation are the main factors that cause muscle atrophy. The treatment of skeletal muscle atrophy has made rapid progress. The current treatment methods are mainly aimed at anti-inflammatory, anti-oxidation, promoting protein synthesis, inhibiting protein degradation, and promoting muscle regeneration, thereby preventing or reducing muscle atrophy. These potential therapeutic methods mainly include drug treatment, gene therapy, stem cell and exosome therapy, cytokine therapy, physical therapy, nutrition support, and other therapies (Figure 3).

### 4.1. Drug Therapy

Currently, the drugs commonly used to treat skeletal muscle atrophy mainly include active substances of traditional Chinese Medicine, chemical drugs, antioxidants, hormone drugs, enzyme, or enzyme inhibitors (Table 1).

#### 4.1.1. Active Substances of Traditional Chinese Medicine

The main hydrophilic component of Salvia miltiorrhiza is magnesium lithospermate B. In high fat diet-induced obese mouse models, administration of magnesium lithospermate B could prevent obesity-related skeletal muscle atrophy by inhibiting MAFbx and MuRF1-mediated muscle degeneration [20]. Puerarin is a flavonoid isoflavone extracted from pueraria, which can reduce hyperglycemia and various diabetes-associated complications. After 8 weeks of oral administration of puerarin, the muscle strength and body mass of type I diabetes mellitus rats were enhanced, and the cross-sectional area of the skeletal muscle was also enlarged, which may be closely related to the fact that puerarin can activate AKT/mTOR and inhibit autophagy [21,22]. Tetramethylpyrazine is the main active component of Ligusticum wallichii Franchat. ITetramethylpyrazine may also ameliorate the disuse-induced loss of muscle mass by inhibiting calcium overload and reactive oxygen species (ROS)-mediated proteolysis and apoptosis [23]. *Morus alba* L., an effective traditional Chinese medicine or beneficial medical supplement, has been widely used to control blood glucose [56]. *Morus alba* L. could increase the levels of p-AMPK and PGC-1α in skeletal muscle and significantly improve insulin resistance and mitochondrial dysfunction in skeletal muscle and L6 myocytes of db/db mice through the AMPK-PGC-1α signaling pathway [24]. Ginsenosides, the main active compounds in ginseng, can prevent memory loss, and they have anti-inflammatory, antioxidant, anti-diabetes mellitus, and anti-tumor effects [25,57]. In Drosophila melanogasters and dexamethasone-treated C2C12 myotube atrophy models, 20(*S*)-ginsenoside Rg3 has been confirmed to promote myoblast differentiation and protect myotube atrophy by regulating the AKT/mTOR/FoxO3 pathway [58,59]. In lipopolysaccharide-treated C2C12 myotubes, triptolide up-regulated protein synthesis signals (IGF-1/Akt/mTOR) and down-regulated protein degradation signal. Triptolide prevented LPS-induced inflammation and skeletal muscle atrophy and might be a novel agent for preventing muscle wasting [27]. Salidroside acts as a bioactive component of Rhodiola rosea and has various functions, such as anti-inflammation, antioxidant, and anti-apoptosis. In a denervation-induced muscle atrophy model, salidroside inhibited the overproduction of ROS and pro-inflammatory cytokines (IL-6), decreased the expression of Foxo3A (a major mediator of skeletal muscle atrophy), inhibited the activation of UPS and ALP, which alleviated muscle atrophy [28,29]. Baicalin is a flavonoid glycoside extracted from Scutellaria baicalensis with anti-apoptotic and anti-inflammatory effects. It can effectively reverse mitochondrial dysfunction, decrease the expression of Cytochrome-c and apoptosis-inducing factors, and activate caspase-3 and caspase-9, thereby inhibiting apoptosis in C2C12 myoblasts and protecting skeletal muscle from injury [31]. Atractylenolide I is a natural sesquiterpene lactone isolated from Atractylodes macrocephala Koidz, inhibited the phosphorylation of STAT3 and PKM2, leading to the downregulation of glycolysis effect and p-SNAP23 expression, reduced EV secretion, thus attenuating muscle wasting and adipose degradation [60]. Corylifol A is one of the active ingredients isolated from *Psoralea corylifolia* L. and has a strong ability of myogenesis [61]. Corylifol A reduces the expression of muscle-specific E3 ubiquitin ligases (MAFbx and MuRF1) and MSTN while activating AKT and protecting myotubes from dexamethasone-induced injury [32]. To conclude, the active substances of traditional Chinese medicine mainly prevent and treat muscle atrophy through anti-inflammatory and antioxidant mechanisms.

#### 4.1.2. Chemical Drugs

IL-6/JAK/STAT3 signaling pathway activated in denervation-induced skeletal muscle atrophy [62,63]. Inhibition of IL-6/JAK/STAT3 signaling pathway by tocilizumab (an anti-IL-6 receptor antibody), ruxolitinib (a JAK1/2 inhibitor), or C188-9 (a STAT3 inhibitor) can suppress proteolysis to alleviate skeletal muscle atrophy [33]. Metformin is a widely used drug for type 2 diabetes mellitus, which can maintain redox homeostasis and induce few side effects [64]. Continuous intraperitoneal injection of metformin for 15 days can alleviate skeletal muscle atrophy in grx1 ko mice, reduce intramuscular lipid sediments, and increase glucose utilization through the AMPK/Sirt1 pathway [34]. Lithium chloride has been shown to enhance muscle differentiation in vitro and prevent muscular dystrophy [35]. In an in vitro and in vivo experiment, lithium chloride exerted therapeutic effects on inflammation-mediated skeletal muscle wasting, such as sepsis-induced muscle atrophy and cancer cachexia [36]. Malotilate, a clinically safe drug and 5-lipoxygenase inhibitor, prevents skeletal muscle atrophy by targeting organogenesis signalling and insulin-like growth factor-1 [65]. Imidazolo-oxindole is a chemical substance that inhibits the autophosphorylation and activity of dependent kinases. It can attenuate LPS-induced inflammation and MuRF1 expression in mouse skeletal muscle, and can alleviate skeletal muscle atrophy by promoting protein synthesis through the AKT/mTOR/S6K1 axis in C2C12 myotubes [37,66]. To conclude, the chemical drugs mainly prevent muscle atrophy through anti-inflammation.

#### 4.1.3. Antioxidants

Damage to the antioxidant defense system can increase ROS production, which further causes mitochondrial dysfunction and apoptosis [67]. Excessive ROS can also up-regulate the expression of IL-6, TNF-α, and TGF-β, acting on MSTN to unbalance protein synthesis and degradation and eventually leading to skeletal muscle atrophy [23,68]. Thus, antioxidants may have great potential as adjunctive therapy against injury, chronic inflammation, or oxidative stress. For example, melatonin, coenzyme Q10, creatine, vitamin D, and vitamin E have been shown to effectively prevent skeletal muscle atrophy [69,70]. Polyphenols are potent natural antioxidants that significantly inhibit oxidative stress and inflammation, thereby reversing mitochondrial dysfunction [71]. Resveratrol prevents sarcopenia by reversing mitochondrial dysfunction and oxidative stress through the PKA/LKB1/AMPK pathway [38]. Carnosol is a bioactive diterpene compound in Lamiaceae plants, which has antioxidant, anti-inflammatory, and anticancer properties. It mainly exerts anti-cachexia effects by inhibiting the TNF-α/NF-κB pathway and reducing muscle and fat loss [39]. Isoquercetin is a bioactive flavonoid with antioxidant and anti-inflammatory properties. Our previous study showed that ROS production decreased in denervated skeletal muscle after treatment with isoquercetin, which may be related to the up-regulation of antioxidant factors (SOD1, SOD2, NRF2, NQO1, and HO1) and the down-regulation of ROS production-related factor (Nox2), and isoquercetin also reversed slow-to-fast fiber type transition after denervation to alleviate target muscle atrophy and mitophagy [9]. In another study, treatment with *N*-acetyl-_L_-cysteine (a clinically used antioxidant) or pyrroloquinoline quinone (a natural antioxidant) could reduce ROS levels in mouse denervated mouse muscle, increase myosin heavy chain and cross-sectional area, and reverse nutrient deprivation-induced C2C12 myotube atrophy [3]. Intraperitoneal injection of pyrroloquinoline quinone (5 mg/kg/d) for 14 days could attenuate denervation-induced mitophagy, fiber-type transition, and skeletal muscle atrophy by inhibiting Jak2/STAT3, TGF-β1/Smad3, JNK/p38 MAPK, and NF-κB signaling pathways [40]. Therefore, antioxidants are important candidates for the prevention and treatment of muscle atrophy.

#### 4.1.4. Hormone Drugs

Hormones are crucial for cell growth and development. Testosterone is an androgenic steroid with a major effect on fat composition and muscle mass of the male body. Androgens attenuate muscle loss and inhibit the slow-to-fast fiber type transition after spinal cord injury in a manner independent of mechanical strain while promoting motor neuron survival. Androgens can increase follistatin expression, decrease MSTN expression, downregulate Acvr2b receptor and Smad 2/3 phosphorylation, inhibit MSTN signaling molecules, and upregulate myogenic markers (MyoD, myogenin, myotube formation, and myonuclei) [72,73]. In the case of cachexia and sarcopenia, exercise is impracticable, and the use of testosterone may be a viable intervention strategy to enhance muscle function. Testosterone in muscle cells can directly stimulate the Ras/MEK/ERK pathway and inhibit MSTN expression [74]. Testosterone also has a potent anti-apoptotic effect in muscle, which inactivates FoxO elements and counteracts H_2_O_2_-induced upregulation of pro-apoptotic genes [41]. Selective androgen receptor modulators are also being developed to avoid various side effects of currently available anabolic steroids [75]. Selective androgen receptor modulators cannot be metabolized into dihydrotestosterone or estrogen, thereby reducing the risk of benign prostatic hyperplasia. However, whether selective androgen receptor modulators can be used in combination with other drugs to treat skeletal muscle atrophy remains to be explored.

Ghrelin is a growth hormone-releasing polypeptide containing 28 amino acids, mainly produced by gastrointestinal tissues, especially the stomach [76]. Ghrelin assists in food absorption and controls the expression of IGF-1 and growth hormone at certain levels to maintain body and muscle mass [77]. However, ghrelin is unfit for medicinal use due to its short half-life. Therefore, anamorelin, which is an orally available and long half-life non-peptide ghrelin mimetic, has been developed [78,79]. Despite their promise, growth hormone-releasing peptides may induce peripheral insulin resistance and diabetes mellitus. Therefore, further research and exploration are still needed.

#### 4.1.5. Enzyme Inhibitors and Activators

##### Cyclooxygenase-2 Inhibitors

Cyclooxygenase exists as two isoforms, namely Cox-1 and Cox-2. Among all these isoenzymes, Cox-2 is the rate-limiting enzyme for prostaglandin synthesis and has been identified as a positive regulator of pathophysiological processes, such as inflammation and oxidative stress. Cox-2 is generally expressed at low or no levels in tissues but is rapidly expressed in response to noxious stimuli or cytokines such as inflammation, oxidative stress, autoimmune responses, mechanical damage, and TGF-β stimulation [80,81]. Aspirin is an inhibitor of Cox-1 and Cox-2 and can effectively relieve denervation-induced skeletal muscle atrophy via the intraperitoneal approach. Aspirin can relieve denervation-induced skeletal muscle atrophy by regulating STAT3 inflammatory signaling pathway and Sirt1/PGC-1α signaling axis, and it can suppress slow-to-fast fiber type transition and mitophagy [43]. Celecoxib is a selective inhibitor of Cox-2. Celecoxib can inhibit denervation-induced skeletal muscle inflammation and oxidative stress, thus controlling the autophagy-lysosomal hydrolysis system and proteasome hydrolysis system, improving blood flow to target muscles, and ultimately alleviating denervation-induced skeletal muscle atrophy [44]. Meloxicam can attenuate lipopolysaccharide-induced protein catabolism in rat muscle by inhibiting the expression of Cox-2, MAFbx, and MuRF1 [82]. Existing evidence has shown that blocking Cox-2 can inhibit fiber formation after injury-induced skeletal muscle atrophy and can inhibit AKT signaling by inhibiting upstream PDK1 expression and impeding TRAF4 recruitment to AKT [83]. Therefore, the Cox-2/PDK1/AKT signaling pathway is expected to become a target for the treatment of skeletal muscle atrophy. Celecoxib treatment has been reported to increase SMN protein levels, improve motor function, and increase survival in a mouse model of severe SMA [84]. After anterior cruciate ligament reconstruction, Celecoxib can effectively reduce quadriceps atrophy while inducing a small increase in C-reactive protein level [85]. Overall, Cox-2 inhibitors can effectively relieve skeletal muscle atrophy, which are important candidates for the treatment of muscle atrophy in clinical practice.

##### Histone Deacetylase Inhibitors

Maintaining the homeostasis between histone acetyltransferases and histone deacetylases is critical for maintaining muscle mass. Under atrophic conditions, this process is disturbed, which activates proteolytic machinery and leads to the degradation of muscle-specific proteins [86]. Suberoylanilide hydroxamic acid (SAHA) as a histone deacetylase inhibitor has become a candidate drug for the treatment of SMA. SAHA is more effective than valproic acid and has been commonly used in clinical practice. SAHA treatment significantly increased SMN levels and vascular density in a SMA mouse model, suggesting that vascular defects in SMA mice could be treated with SAHA [87]. Trichostatin A is also a well-known class I and class II histone deacetylase inhibitor. It activates SMN2 gene expression in vivo and ameliorates SMA phenotypes when administered after onset [47]. Studies have shown that trichostatin A can treat C2C12 myotube atrophy caused by nutritional deficiency and inactivate FoxO by reducing HDAC4 activity and myogenin levels, thus alleviating denervation-induced skeletal muscle atrophy [86,88]. Trichostatin A can inhibit cigarette smoke exposure-induced skeletal muscle atrophy by downregulating the markers of UPS, apoptosis, and pyroptosis via HDAC1/2 inhibition [45]. Trichostatin A inhibits unloading-induced soleus muscle atrophy through MuRF1 regulation, partially suppressing the decline in type I and type IIa fibers in the soleus muscle and reversing the transition from slow-twitch to fast-twitch muscle fibers [48]. Continuous injection of HDAC1 inhibitor CI-994 (1 mg/kg/day) for 3 days in male Wister rats suspended from the hindlimb significantly inhibited the decrease and atrophy of actin content in the soleus muscle of rats [89]. In conclusion, Histone deacetylase inhibitor can be used as a candidate drug in preventing or reducing skeletal muscle atrophy.

##### Phosphodiesterase Inhibitors

Phosphodiesterase (PDE) is an intracellular enzyme that can degrade cAMP and/or cGMP. The use of PDE inhibitors is very promising in modulating multiple pathophysiological conditions including skeletal muscle atrophy. Rolipram and roflumilast, two PDE-selective inhibitors, have been confirmed to alleviate skeletal muscle atrophy in animal models of chronic diseases. Rolipram can reduce the levels of MAFbx and MuRF1 in the soleus muscle and extensor digitorum longus and reduce the activity of calpain and caspase-3 to treat skeletal muscle atrophy in diabetes mellitus rats [49]. In myotubes of patients with cachexia and chronic obstructive pulmonary disease, roflumilast improves the expression of slow myosin and fast myosin isoforms, increases cAMP signal, and reduces UPS and MSTN gene expression via NRF2 upregulation to promote antioxidant defense pathways and histone deacetylase sirtuin-1 pathway, thus treating muscle dysfunction and atrophy [50]. Torbafylline (HWA 448) and pentoxifylline are two non-selective PDE inhibitor xanthine derivatives that help prevent skeletal muscle atrophy induced by cancer, sepsis, trauma, sepsis, chronic obstructive pulmonary disease, and fasting. Torbafylline reduces inflammatory cytokine levels in skeletal muscle atrophy caused by injury, denervation, diabetes mellitus, cancer, and sepsis. In animal models, pentoxifylline administration downregulates Ca^2+^-dependent calpain and Ca^2+^-independent Cathepsin L, stimulates cAMP formation, and reduces the overall rate of protein degradation. Burn increases muscle PDE4 activity and proteolysis and decreases cAMP levels. Torbafylline can reverse the burn-induced upregulation of PDE4 activity; decrease the cAMP level; and increase the levels of TNFα, IL-6, ubiquitin, and E3 ligase in rat skeletal muscle, and it can then activate the PDE4/cAMP/Epac/PI3K/AKT pathway to inhibit burn-induced muscle proteolysis in the rat model of burn [51]. Pentoxifylline also mitigates the loss of muscle mass under catabolic conditions, primarily by inhibiting protein degradation. In a rat model of diabetes mellitus, pentoxifylline could activate cAMP and AKT and inhibit the expression of atrogenes and calpain/caspase-3, thus relieving muscle atrophy [52]. In conclusion, phosphodiesterase inhibitors can effectively inhibit muscle atrophy and act as candidate drugs for the prevention and treatment of muscle atrophy.

##### m6A Demethylase ALKBH5

Alpha-ketoglutarate-dependent dioxygenase alkB homolog 5 (ALKBH5) is a major m6A demethylase, and the potential regulatory mechanisms of ALKBH5 that depend upon m6A modification are related to long non-coding RNA, autophagy, and hypoxia [53,54]. Recent studies have found that ALKBH5, an m6A demethylase, can control adult muscle mass, and m6A is a regulator of muscle health and diseases. The overexpression of ALKBH5 reduces m6A levels and further activates FoxO3 signaling, thus inducing an excessive reduction in muscle mass. ALKBH5 demethylates HDAC4 mRNA and stabilizes HDAC4 mRNA. HDAC4 interacts with and deacetylates FoxO3, resulting in a marked increase in FoxO3 expression and activity, whereas the specific deletion of ALKBH5 impedes FoxO3 activation and denervation-induced skeletal muscle atrophy in the mouse model [55]. These results indicate that ALKBH5 is a potential therapeutic target for neurogenic muscle atrophy.

### 4.2. Gene Therapy

#### 4.2.1. Gene Medicine

Duchenne muscular dystrophy (DMD) is an X-linked inherited neuromuscular disorder due to mutations in the DMD gene coding for dystrophin. DMD is characterized by progressive skeletal muscle atrophy and myocardial weakening, ultimately inducing death. Existing evidence has shown that patients with DMD can live up to their 30 s with the assistance of ventilators and intensive care [90]. FDA-approved ASO drugs for DMD include eteplirsen (EXONDYS 51^®^), golodirsen (VYONDYS 53™), and viltolarsen (VLTEPSO^®^) [91]. Ataluren (Translarna™) was the first FDA-approved drug for DMD, primarily for use in patients with nonsense mutation DMD aged 2–5 years [92]. In 2016, eteplirsen was FDA-approved for treating DMD with mutations in the dystrophin gene by inducing exon 51 skipping to promote the production of functional dystrophin [93]. In 2019 and 2020, golodirsen and viltolarsen were also approved for the treatment of DMD patients with a confirmed mutation in the dystrophin gene that is amendable to exon 53 skipping [91,94,95]. Viltolarsen is the first and only exon 53-skipping therapy to increase dystrophin levels in children at the age of 4 years or less [95,96]. The large size of the dystrophin gene is the major limitation for the use of gene therapy in the treatment of DMD. Current treatment regimens generally applicable to all DMD types remain limited in their potential benefits and are likely not to specifically target dystrophin upregulation. Therefore, in-depth research in this field is still required.

#### 4.2.2. Gene Overexpression and Knockdown

Muscle atrophy can be treated via the knockdown of muscle atrophy-related genes. A recent study showed that NLRP3 knockout protected mice from sepsis-induced skeletal muscle atrophy [97]. The knockdown of HDAC4 inhibits the conversion of type I fibers to type II fibers by reducing the expression of MYOG, muscle-specific E3 ubiquitin ligases (MuRF1 and MAFbx), and autophagy-related proteins (Atg7, LC3B, PINK1, and BNIP3), and it further enhances the expression of SIRT1 and PGC-1α to alleviate denervation-induced skeletal muscle atrophy. This provides a theoretical basis for the study of potential drugs for neurogenic skeletal muscle atrophy [19]. The knockdown of TRAF6 attenuates dexamethasone-induced skeletal muscle atrophy [4]. TRAF6-shRNA transfected into nutrient-deprived C2C12 myotubes or injected into the tibialis anterior muscle of denervated mice can simultaneously reduce the expression of MuRF1 and MAFbx, thereby blunting the reduction of the myotube diameter and the loss of tibialis anterior muscle mass [98]. Activated STAT3 (pSTAT3) stimulates MSTN through C/EBPδ to initiate skeletal muscle atrophy. The knockdown of STAT3 or C/EBPδ or MSTN may serve as therapeutic targets for skeletal muscle atrophy [33,99]. The knockdown of these key molecules can indeed alleviate skeletal muscle atrophy, but the specific molecular mechanisms remain to be further explored in order to provide scientific evidence for future clinical treatments.

SIRT1 is a member of the sirtuin family of class III nicotinamide adenine dinucleotide-dependent protein deacetylases. The knockout of SIRT1 in satellite cells impairs muscle function, and the overexpression of SIRT1 increases muscle repair and muscle fatigue resistance in aged mice [100]. During intermittent fasting, SIRT1 can also suppress type I fiber atrophy by deacetylating and inhibiting the transcriptional activity of FoxO1 and FoxO3 [101]. JunB is a transcription factor that promotes cell division. When JunB is transfected into denervated muscle, it blocks FoxO3 binding to the MAFbx and MuRF1 promoters and inhibits MAFbx and MuRF1 expression, thereby reducing proteolysis and preventing muscle fiber atrophy [102]. The overexpression of the mitochondrial transcription factor A (TFAM) gene reduces both soleus and gastrocnemius muscle atrophy that occurs during hindlimb suspension [103]. TFAM is also able to protect mtDNA from ROS degradation and initiate mitochondrial protein transcription while improving mitochondrial function [104]. The overexpression of PGC-1α can alleviate the effects of aging on Fis-1 and Drp-1 expression, enhance mitochondrial oxidative function and antioxidant enzyme activity, reduce lipid peroxidation and endomembrane damage, effectively improve mitochondrial defects, and reduce muscle fiber atrophy in aged mice [105,106]. PGC-1α can also induce myonuclear hyperplasia and reduce myonuclear domain (MND) volume via sufficient transcription and the synthesis of mitochondria-associated proteins [107]. Mitophagy is associated with the degree of skeletal muscle atrophy. STAT3 knockdown significantly inhibits mitophagy, manifested as decreased numbers of autophagic cells and autophagic vesicles, as well as dramatically the decreased expression of autophagy-related factors PINK1, BNIP3, LC3B, ATG7, and Beclin 1 [33]. To conclude, there is an emerging therapeutic strategy for muscle atrophy, which targets the key regulatory molecules in the process of muscle atrophy via knockdown or overexpression, thus improving the target muscle microenvironment, inhibiting proteolysis, promoting protein synthesis, and improving mitochondrial function. However, its specific molecular regulation mechanism and biological safety still need to be further explored.

#### 4.2.3. Non-Coding RNAs (ncRNAs)

In the literature, miRNAs have been identified as important biomarkers with important implications for the diagnosis and treatment of various diseases [108]. Freire et al. revealed that miR-497-5p may be involved in a compensatory mechanism in response to IL-6-induced skeletal muscle atrophy [109]. The bone marrow-derived miR-223-3p represses the target gene IL-6 [110]. These two miRNAs modulate the initial immune response, rescue impaired regenerative capacity, and reduce fibrosis. In sepsis mice, miR-140 inhibits endotoxin-induced glycolysis and atrophy of skeletal muscle by negatively regulating the WNT signaling pathway and simultaneously reducing the expression of Wnt family member 11, β-catenin, and GSK-3β [111]. After type 2 diabetes mellitus, miR-193b induces the inactivation of the Akt/mTOR/S6K pathway, reducing protein synthesis and muscle mass and providing a new miRNA therapeutic target for muscle regeneration in degenerative muscle diseases [112]. With anti-fibrotic activity, miR-29 attenuates skeletal muscle atrophy in chronic kidney disease (CKD) and miR-26a restricts skeletal muscle atrophy through exosome-mediated miRNA transfer [113,114]. In denervation-induced muscle atrophy, miR-125b-5p targets TRAF6 to inhibit the expression of UPS and ALP-related proteins, thus reversing the atrophy of rat tibialis anterior muscle and C2C12 myotubes [98]. miR-351 significantly inhibits dexamethasone-induced reduction in C2C12 myotube diameter and denervation-induced muscle atrophy through the negative regulation of TRAF6 and two downstream signaling molecules of TRAF6, MuRF1 and MAFbx [115,116]. In muscle atrophy caused by denervation, injury, diabetes mellitus, and CKD, these miRNAs play a role in the treatment of skeletal muscle atrophy through different molecular mechanisms. Further in-depth studies of these miRNAs can indeed provide new therapeutic strategies for skeletal muscle atrophy.

In addition to miRNAs, circRNAs and lncRNAs also play important roles in skeletal muscle atrophy. CircRNAs are associated with a variety of biological processes. circCCDC91 has potential functions in chicken skeletal muscle development. circCCDC91 can absorb the miR-15 family (miR-15a, miR-15b-5p, and miR-15c-5p) to regulate IRS-1 expression and activate the IGF1-PI3K/AKT signaling pathway, promote myoblast proliferation and differentiation, and alleviate skeletal muscle atrophy [117]. The lncRNAs, acting as ceRNAs, can exert vital roles in gene expression regulation [118]. lncIRS1 has been used as a ceRNA of the miR-15 family to regulate the expression of IRS-1. The overexpression of lncIRS1 not only increases the protein abundance of IRS-1, but also promotes the level of p-AKT, a core component of the IGF1-PI3K/AKT pathway; regulates the expression of atrophy-related genes; and reverses skeletal muscle atrophy [119]. However, the specific roles of circRNAs and lncRNAs in skeletal muscle atrophy remain to be further investigated.

### 4.3. Stem Cell and Exosome Therapy

Currently, cell therapy is one of the most promising treatment methods. Stem cells and stem cell-derived exosomes have been used to treat muscle atrophy, and this approach has become a research hotspot (Table 2).

#### 4.3.1. Muscle-Derived Stem Cells (MDSCs)

MDSCs can be expanded in vitro up to 30 passages while maintaining myogenic potential, with great promise in the treatment of various diseases, such as sarcopenia and diabetes mellitus [140,141]. CD133^+^ angioblasts, human skeletal muscle pericytes, and satellite cells are the most commonly studied MDSCs in skeletal muscle. CD133^+^ angioblasts and pericytes are closely associated with blood vessels in tissues, such as skeletal muscle, and play a key role in tissue homeostasis. In a mouse model of cryoinjury, intramuscular transplantation of myogenic CD133^+^ cells repopulated satellite cell niches, and these donor cells were observed to generate an efficient regenerative response following subsequent reinjury [122]. Dysferlin is abundantly expressed in skeletal and cardiac muscles, and its main function is membrane repair. Transplantation of engineered blood-derived CD133^+^ stem cells into immune/dysferlin-deficient scid/blAJ mice could induce sufficient dysferlin expression to correct functional deficits during skeletal muscle membrane repair [142]. In the case of muscle injury, pericytes contribute to generating a regenerative microenvironment by releasing trophic factors and modulating local immune responses [143]. These cells also maintain potency via in vitro expansion and spontaneously differentiate into multinucleated myotubes [124]. Although satellite cells account for only a small fraction (2–7%) of the muscle cell population, the transplanted cells can produce a large number of offspring cells and form muscle fibers to transfer into the defective area, thus improving the contractile function of the host muscle [120]. However, satellite cells are more difficult than MDSCs to isolate, purify, survive, and massively expand in vitro. Therefore, MDSCs may be a better choice for the treatment of muscle atrophy.

#### 4.3.2. Non-Muscle-Derived Stem Cells

Non-myogenic stem cells mainly include mesenchymal stem cells (MSCs), induced pluripotent stem cells (iPSCs), and embryonic stem cells (ESCs).

MSCs possessing multilineage differentiation potentials can be isolated from various tissues, including adipose tissue, bone marrow, cranial neural crest, tonsils, umbilical cord, and allogeneic placenta [128,144]. In muscle injury models, MSCs from different sources can improve muscle contractile function, reduce scar tissue, and increase muscle fiber formation and blood vessel density. MSCs also secrete multiple cytokines, including growth factors, that promote angiogenesis, cell recruitment, migration, proliferation, and differentiation [145]. Bone marrow-derived MSCs (BM-MSCs), allogeneic placenta-derived MSCs (PL-MSCs), and umbilical cord-derived MSCs (UC-MSCs) have become issues of concern. The intramuscular and intraarterial transplantation of autologous BM-MSCs improves muscle contractile function after a severe crush injury or sphincter injury [126,146]. Allogeneic PL-MSCs have been confirmed to reduce fibrosis and inflammation in mdx mice, a model animal for DMD [128]. For treating dexamethasone-induced muscle atrophy, UC-MSCs can reduce the expression of skeletal muscle atrophy-related proteins and make the expression of muscle-specific proteins and oxidase close to normal, and they can effectively inhibit the production of ROS [129]. Meanwhile, MSC transplantation is a promising approach to alleviate age-related sarcopenia [147,148]. IPSCs and ESCs have the unlimited ability to proliferate, self-renew, and differentiate into myogenic progenitor cells and myotubes, which have become useful tools for drug screening and personalized medicine in clinical practice, as well as a better choice for the treatment of degenerative diseases [149]. Patient-derived iPSCs and ESCs are ideal cell sources that have no immune rejection after transplantation. Current approaches for inducing ESCs/iPSCs into skeletal muscle cells include the overexpression of muscle-associated transcription factors (eg., MyoD, Pax3) or the addition of small molecules, activation of myogenic signals to generate mesoderm cells during development, or induction of myogenic progenitor cell regeneration to help restore lost muscle fibers [131,132]. Recently, iPSCs have been found to help design myogenic phenotypes, provide biomimetic manufacturing, and play a certain role in skeletal muscle tissue engineering [133]. The above findings still need to be further explored, in order to provide a new insight into the treatment of muscle atrophy.

#### 4.3.3. Exosomes

Exosomes are a natural carrier system that transfers nucleic acids, proteins, lipids, and cellular signals between donor and recipient cells through autocrine, paracrine, and endocrine patterns and thus remodels the extracellular matrix, and the exosomes can serve as a therapeutic agent in various disease models [135,150,151,152]. Lamp2b is an exosome membrane protein gene fused with a muscle-specific surface peptide for muscle delivery. A Lamp2b-containing exosome vector is transfected into muscle satellite cells, and transfected cells are then transduced with adenovirus expressing miR-26a to generate exosomes-encapsulated miR-26a (Exo/miR-26a), which can be used for the treatment of CKD mice by increasing the cross-sectional area of skeletal muscle [114,135]. Exo/miR29 can ameliorate skeletal muscle atrophy and alleviate renal fibrosis by downregulating YY1, a pro-fibrotic protein in the kidney, and TGF-β pathway proteins [134,135]. In addition to muscle atrophy caused by chronic diseases, exosomes can also be used to treat disuse muscle atrophy. miR-421/FOXO3a is a direct target of circHIPK3. Exosomes from UC-MSCs downregulate miR-421 by releasing circHIPK3 and thus increase the expression of FOXO3a, thereby inhibiting cell pyroptosis and release of IL-1β and IL-18 to prevent ischemic skeletal muscle injury [136]. The treatment of myoblasts with exosomes from PL-MSCs increases the differentiation of these cells and decreases the expression of fibrotic genes in myoblasts from DMD patients [128]. Exosomes from differentiated human skeletal myoblasts are rich in various myogenic factors: TNF, IGFs, and FGF2. These exosomes can induce myogenic differentiation of human adipose-derived stem cells and increase the fusion index and expression of myogenic genes (ACTA1, MYOD1, DAG1, DES, TNNT1, and MYH1/2) in the cells, leading to skeletal myogenesis [137]. Furthermore, exosomes from human BM-MSCs can promote the proliferation and differentiation of C2C12 cells [153]. Extracellular vesicles derived from skin precursor-derived Schwann cells (SKP-SC-EVs) contain a large number of antioxidants and anti-inflammatory factors. In vitro and in vivo studies have found that SKP-SC-EVs reduce the levels of ROS, IL-1β, IL-6, and TNF-α in nutrient deprivation-induced myotubes and muscles, inactivate the activity of ALP, and control proteolysis, thereby alleviating denervation-induced skeletal muscle atrophy [6]. After the treatment with exosomes from human BM-MSCs, the level of miR-486-5p is up-regulated, and the nuclear translocation of FoxO1 is inhibited, thereby slowing dexamethasone-induced muscle atrophy [139]. The specific silencing of atrophic skeletal muscle fibre-derived small extracellular vesicle miR-690 in the muscle can promote satellite cell differentiation and alleviate muscle atrophy in aged mice [154]. Exosomes from different states of cells have unique contents and exert unique effects [135]. Therefore, engineered extracellular vesicles (EVs) should be a promising issue. It can transmit signal molecules in vesicles directly to muscle cells, which may become a new drug for the treatment of muscle atrophy.

### 4.4. Cytokines

Growth factors are a class of cytokines that stimulate cell growth. Common growth factors for treating skeletal muscle atrophy include vascular endothelial growth factor, hepatocyte growth factor, fibroblast growth factor (FGF), and human epidermal growth factor. Sustained delivery of vascular endothelial growth factors can promote angiogenesis and muscle formation [155]. Rapid release of hepatocyte growth factor loaded on fibrin microfilament scaffolds promotes functional muscle tissue remodeling and enhances skeletal muscle regeneration in a mouse model [156]. FGF19 is an endocrine-derived hormone that has recently emerged as a potential target for the treatment of metabolic diseases. FGF19 improves grip strength and muscle atrophy in young and old mice given a high-fat diet. FGF19 abrogates the increase in the markers of skeletal muscle atrophy (FOXO-3, MAFbx, MuRF1) in palmitic acid-treated C2C12 myotubes and skeletal muscle in high-fat diet-fed mice, which can reduce obesity-induced skeletal muscle atrophy through the AMPK/SIRT-1/PGC-α signaling pathway [157]. FGF19 can ameliorate skeletal muscle atrophy induced by glucocorticoid treatment or obesity, which has been proved in a mouse model [158]. The combination treatment of adipose-derived stem cells and basic fibroblast growth factor with hydrogel as a carrier can achieve revascularization and reinnervation and reduce fibrosis in torn muscles [159]. Emerging skeletal muscle tissue engineering technology attempts to construct exogenous muscle tissue to treat volumetric muscle loss, and human epidermal growth factor delivered at optimal concentration and delivery time can enhance in vitro skeletal muscle cell proliferation and differentiation during myogenesis, providing a new idea for the treatment of muscle atrophy [160].

IGF-1 is a 7.5 kDa polypeptide that is structurally related to insulin. It is a circulating hormone secreted by the liver in response to pituitary growth hormone, but it is also an autocrine factor released by muscle fibers. IGF-1 can regulate protein synthesis in skeletal muscle and promote body growth and has also been shown to activate satellite cell proliferation [161]. Existing evidence indicates that IGF-1/AKT can inhibit skeletal muscle atrophy-inducing factors and MSTN signaling transduction by inhibiting NF-κB and Smad pathways, respectively [18]. Local overexpression of IGF-1 has successfully rescued various chronic and experimental skeletal muscle atrophy, including the injection of dexamethasone, aging, hindlimb suspension, ALS, and Duchenne muscular dystrophy [162]. Many biological pathways regulated by IGF-1 can interact with each other, which limits the study of IGF-1 in skeletal muscle. Any treatment that increases IGF-1 levels may increase the risk of tumor formation or the growth of an existing cancer. Therefore, IGF-1 is still worthy of further exploration in the treatment of muscle atrophy.

In addition to growth factors, some other cytokines also play an important role in the repair of skeletal muscle atrophy, such as stromal cell-derived factor-1 (SDF-1), pigment epithelium-derived factor (PEDF), bone morphogenetic protein-7, FilaminC, and Irisin. SDF-1 is the sole ligand for CXCR4 and is involved in skeletal muscle development. In chicken embryos, overexpression of SDF-1 can enhance the SDF-1/CXCR4 signaling pathway to induce somatic cell proliferation. Somatic cells express most SDF-1 in the limb, which promotes the increase in limb blood vessels. This indicates that SDF-1 promotes the proliferation of myogenic and vascular-derived progenitor cells, thus controlling the formation of limb muscles and blood vessels [163]. SDF-1a can act on adipose-derived stem cells to improve muscle structure and function, reduce fibrosis development, and modulate immune response [164]. PEDF-derived short peptide is a derivative of PEDF and can induce the phosphorylation of ERK1/2, AKT, and STAT3 in C2C12 myoblasts. PEDF/PEDF-derived short peptide stimulates the proliferation of rat muscle fiber primary satellite cells in vitro while increasing the expression of cyclin D1. The delivery of PEDF-derived short peptide can stimulate the proliferation of satellite cells and promote the growth of regenerating muscle fibers in damaged muscle [165]. Bone morphogenetic protein-7 is an osteoporosis drug, which can target and inhibit cell pyroptosis and improve muscle function of diabetes mellitus. Therefore, bone morphogenetic protein-7 may be a potential treatment option for diabetes mellitus-induced myopathy [166]. FilaminC plays an active regulatory role in myoblast development. FilaminC interacts with Disheveled-2 to activate the wnt/β-catenin signaling pathway and control skeletal muscle development [167]. Irisin is a skeletal muscle-secreted myokine that can be delivered into the circulation by cleavage of fibronectin type III domain containing protein 5 (FNDC5). Exercises can induce Irisin expression [168]. Studies have shown that Irisin can protect skeletal muscles from denervation-induced atrophy, and it promotes myogenic differentiation and the fusion of myoblasts by activating IL-6 signaling, thereby promoting muscle regeneration. Furthermore, it rescues the loss of skeletal muscle mass after denervation by enhancing satellite cell activation and reducing protein degradation [169]. These cytokines can be used as potential targets for the treatment of skeletal muscle atrophy, but their biological safety remains to be further studied.

### 4.5. Physical Therapy

#### 4.5.1. Electrical Stimulation and Optogenetic Technology

Methods using electrical current to alter neuromuscular activity include neuromuscular electrical stimulation, transcutaneous electrical nerve stimulation, and functional electrical stimulation (FES). Among them, FES is the focus of the current research on restoring muscle function in paralyzed patients. Electrical stimulation of the hypoglossal nerve has been shown to prevent tongue relaxation and subsequent airway closure in patients with obstructive sleep apnea [170]. FES-cycling and resistance training can slow muscle atrophy induced by spinal cord injury or promote muscle hypertrophy [171]. Blood flow restriction combined with electrical muscle stimulation represents an effective intervention strategy to mitigate the loss of muscle mass during limb disuse but cannot implicate strength maintenance [172]. Neuromuscular electrical stimulation is safe, practical, and effective for improving functional capacity and muscle strength in hemodialytic patients. However, further studies are needed to confirm the clinical relevance of these findings [173]. Direct electrical stimulation of muscle fibers requires a large amount of energy, which can produce toxic gases and trigger nerve damage [174]. Although indirect electrical stimulation reduces energy and side effects, afferent sensory nerves may still be affected [175]. Electrical stimulation can maintain muscle mass and maximize force production. Unfortunately, when peripheral nerves are damaged or dysfunctional, such as denervation of skeletal muscles leading to atrophy, electrical stimulation is often invalid for motor function and even stimulates nearby sensory nerves to produce unpleasant sensations (pain).

Channelrhodopsin-2 (ChR2) is a light-driven cation channel from the green algae/Chlamydomonas reinhardtii, acting as an optogenetic tool. Routine stimulation of ChR2-expressing hindlimb muscle fibers within the sarcolemma and T-tubules by percutaneous irradiation is sufficient to reduce denervation-induced muscle atrophy and maintain muscle contractility even after sciatic nerve injury [176]. To recover muscle motor function, photosensitive actuators with different spectral sensitivities are used, which are specifically expressed in different types of muscle fibers, such as slow-twitch and fast-twitch fibers, allowing the precise control of muscle contraction by mimicking physiological patterns [177]. Optogenetic stimulation to restore muscle health is highly sought after for its particular advantages. It includes both indirect optogenetic stimulation of innervating nerves of FES and direct optogenetic stimulation of skeletal muscle [178]. Optogenetic stimulation has been proposed as an alternative to overcome some shortcomings of FES, enabling specific, spatially and temporally precise stimulation of ChR2-expressing cells [179,180]. The clinical translation of optogenetic stimulation requires overcoming certain hurdles, including successful gene transfer, sustained optogenetic protein expression, and the creation of optically active implantable devices.

#### 4.5.2. Electroacupuncture

Accumulation of chronic exercise injuries can generate the deposition of collagen fibers in skeletal muscle, resulting in skeletal muscle fibrosis. Acupuncture can inhibit skeletal muscle fibrosis by downregulating the TGFβ1/ERK/CTGF signaling pathway [181]. In a rat model of exercise-induced skeletal muscle injury, acupuncture could effectively improve exercise-induced skeletal muscle injury and reduce endoplasmic reticulum stress after heavy-load eccentric exercise. The mechanism may be related to the up-regulation of protein disulfide isomerase and the inhibition of the endoplasmic reticulum stress PERK pathway [182]. Acupuncture plus low-frequency electrical stimulation (Acu-LFES) can achieve the intended results of exercise by stimulating muscle contraction, thereby enhancing muscle regeneration and preventing muscle loss [183]. This combined treatment is suitable for patients with serious diseases who cannot exercise regularly. Acu-LFES counteracts skeletal muscle atrophy induced by diabetes mellitus by increasing IGF-1 and stimulating muscle regeneration [183]. Potential mechanisms by which Acu-LFES ameliorates CKD-induced skeletal muscle atrophy include the activation of M2 macrophages and reversal of MAFbx expression [184]. Electroacupuncture has been shown to inhibit MSTN expression, inducing satellite cell proliferation and skeletal muscle repair [185]. Electroacupuncture reduces myocyte apoptosis and improves denervation-induced muscle atrophy through elevation of p-AKT (Ser473) levels and activation of the AKT signaling pathway [186]. Electroacupuncture combined with massage can alleviate fibrosis by modulating TGFβ1-CTGF-induced myofibroblast transdifferentiation and MMP-1/TIMP-1 balance for extracellular matrix production [187]. In conclusion, electroacupuncture has a good therapeutic effect on skeletal muscle atrophy caused by various factors.

#### 4.5.3. Low-Level Laser Therapy (LLLT)

LLLT is a novel adjunctive intervention that protects cancer patients from doxorubicin-induced skeletal muscle atrophy. Doxorubicin is an anthracycline drug widely used in cancer treatment, which produces many adverse effects. LLLT inhibits doxorubicin-induced mitochondrial dysfunction, apoptosis, and oxidative stress and prevents doxorubicin myotoxicity via AMPK activation and upregulation of SIRT1 and its downstream signal PGC-1α [188]. LLLT can significantly reduce the content of connective tissue in the tibialis anterior muscle of rats with nerve compression injury [189]. Studies have also demonstrated the ability of LLLT to delay the progression of disuse muscle atrophy. Furthermore, LLLT can enhance muscle contraction in healthy control rats [190]. LLLT reduces bupivacaine-induced fibrosis and necrosis of the sternocleidomastoid muscle and accelerates muscle regeneration [191]. LLLT exerts a protective effect against skeletal muscle injury after ischemia/reperfusion injury by inhibiting early inflammatory responses and muscle necrosis and stimulating neovascularization [192]. The above findings suggest that LLLT may be a new auxiliary intervention for the prevention and treatment of muscle atrophy, but the molecular mechanism of its action remains to be further studied.

#### 4.5.4. Heat Therapy (HT)

HT regulates many signaling pathways, including angiogenesis, anabolism, mitochondrial biogenesis, and glucose homeostasis [193,194,195,196]. These lines of evidence suggest that repeated exposure to HT promotes capillary growth and hypertrophy, increases mitochondrial content and function, and alters glucose metabolism and insulin signaling. Mild HT is an effective therapeutic strategy to promote satellite cell proliferation and differentiation into myofibers. In cells transfected with siRNA targeting PGC-1α, mild HT-induced myogenic differentiation and myogenin expression were significantly suppressed, suggesting that mild HT regulates PGC-1α to promote myogenic differentiation [197]. HT can induce a stress response and thus increases heat shock protein expression and improves mitochondrial function, thereby attenuating limb immobilization-induced muscle atrophy [193]. HT alleviates muscle atrophy of extensor digitorum longus in streptozotocin-induced diabetic rats by upregulation of HSP72 and HSP25 [198]. HT can restore the dexamethasone-induced inhibition of PI3K/AKT signaling and reduce the increased expression of REDD1 and KLF15, thereby preventing dexamethasone-induced muscle atrophy [199]. The specific mechanism of HT remains to be further studied.

### 4.6. Nutrition Support

For patients with limited mobility or muscle damage, nutritional support may be a good alternative to slow muscle atrophy. Increasing evidence has shown that proteins, essential amino acids, β-hydroxy-β-methylbutyrate, creatine, vitamin D, and other nutrients are crucial for the treatment of skeletal muscle atrophy [200,201]. Insufficient protein intake cannot meet the daily needs of the human body and results in a negative protein balance, which causes skeletal muscle atrophy and inhibits muscle growth and function. Protein intake has an important role in the treatment of sarcopenia [202]. Branched chain amino acids such as leucine can alleviate the loss of muscle mass [203]. A diet of 18% protein + 3% leucine could improve muscle strength and behaviors, maintain body mass, fat, and muscle mass and decrease some markers of protein degradation in tumor-bearing Wistar rats. In fact, a leucine-rich diet alone does not fully cure cachexia but may potentially reduce muscle protein degradation and promote muscle performance [204]. β-Hydroxy-β-methylbutyrate supplementation may also be a potential nutritional strategy to counteract muscle mass loss. A study recruiting 472 patients with cancer who were supplemented with a mixture of β-hydroxy-β-methylbutyrate, arginine, and glutamine found a decrease in protein breakdown and an increase in protein synthesis, thus reducing muscle atrophy [205]. In older adults, creatine supplementation alone appears to have little benefit in muscle function and mass, but creatine as a supplement to exercise training seems to enhance adaptive muscle responses to training stimuli [201]. Therefore, creatine may be an effective dietary strategy. Vitamin D supports cellular redox homeostasis by maintaining normal mitochondrial function. However, vitamin D supplementation remains controversial to overcome skeletal muscle atrophy. Calcitriol mediates ROS production through PKC, which in turn induces the atrophy of C2C12 myotubes. Co-treatment with the antioxidant NAC is sufficient to blunt the myotube-promoting activity of calcitriol while its upstream metabolites, cholecalciferol (vitamin D3) and calcifediol (25-OH-vitamin D3), have anti-atrophic and antioxidant properties [206]. These findings suggest that the efficacy of vitamin D supplementation may come from the balance between vitamin D metabolites. In addition, hydrolysates of crassostrea gigas have been found to inhibit skeletal muscle atrophy by regulating protein synthesis via the PI3K/AKT/mTOR pathway and by regulating mitochondrial biogenesis via the SIRT1/PGC-1α signaling pathway [207]. A long-term ketogenic diet alleviates aging-induced sarcopenia in mice because it improves mitochondrial function and antioxidant capacity [208]. Dietary supplementation with _L_-carnitine attenuates muscle damage and reduces cellular damage and free radical formation while reducing muscle soreness [209]. Docosahexaenoic acid is a major dietary omega-3 polyunsaturated fatty acid (omega-3 PUFA); both UPS and ALP are regulated by docosahexaenoic acid, which has been shown to delay skeletal muscle atrophy by inhibiting UPS [15,210]. Omega-3 polyunsaturated fatty acids have been shown to reduce the development of sarcopenia in the elderly population via the positive regulation of intracellular metabolic signaling [211]. Therefore, omega-3 polyunsaturated fatty acids may be potential nutrients for the prevention and treatment of muscle atrophy. Studies evaluating the impact of individualized nutritional interventions on muscle mass and clinical outcomes in patients with cancer and sarcopenia are very limited, and randomized, large-scale, and long-term clinical trials are still needed to verify the positive effects of nutritional interventions on muscle metabolism.

### 4.7. Others

In addition to the above treatment methods, there are other unique methods for use in the treatment of skeletal muscle atrophy, such as adjuvant treatment with biomaterials such as hydrogels, intestinal microbial regulation, and oxygen supplementation for muscle atrophy caused by high altitude. Biomaterials have been developed to wrap and protect donor cells. For example, the use of scaffolding technology to encapsulate cells in a protective environment can bypass some limitations of inflammation-related adverse factors and greatly improve therapeutic efficacy while reducing the number of cells required for the treatment [212]. Hydrogels are a class of materials with the unique advantage, which are less invasive and fully degradable in vivo. It is currently being used in conjunction with cell therapy and/or growth factor delivery to facilitate the treatment of muscle damage and skeletal muscle atrophy [213] while accelerating angiogenesis and nerve regeneration [214] and benefiting the structural and functional reconstruction of target muscles. It has been reported that the gut lumen of squirrels contains microorganisms with the urease gene. That is, these microorganisms can metabolize urea into carbon dioxide and ammonium. Ammonium is then used by the same microbiota as a nitrogen source to produce amino acids, and nitrogen losses during protein catabolism and urea formation can be compensated, thereby counteracting muscle atrophy [214]. The contribution of microorganisms to urea nitrogen reuse in hibernating mammals provides a potential target for the development of new therapies for skeletal muscle atrophy and related disorders. In addition, long-term exposure to high altitudes also causes a decrease in muscle mass due to hypoxia. The mechanism by which hypoxia causes skeletal muscle atrophy is not fully understood, but some clinical studies have suggested that supplemental oxygen improves muscle function. Therefore, oxygen supplementation may also be one of the potential treatments for muscle atrophy.

## 5. Conclusions and Prospects

The mechanisms underlying skeletal muscle atrophy, defined as a reduction of muscle mass, are complex. Growing evidence suggests that major protein catabolism pathways, including the ubiquitin-proteasome system, autophagic-lysosomal pathway, and calpains systems as well as protein synthesis, are disturbed in skeletal muscle atrophy. Oxidative damage and inflammation are potential factors contributes to muscle atrophy. The current treatments and strategies for skeletal muscle atrophy mainly aimed at above factors including drug treatment, gene therapy, stem cell and exosome therapy, cytokine therapy, physical therapy, nutrition support, and other therapies. Stem cell and exosome therapies have the definite potential to treat traumatic skeletal muscle injury, but overcoming existing limitations and optimizing variables will be the key to clinical translation. Personalized nutritional interventions are friendly to those who are sick in bed. Although various kinds of drugs have been developed, the molecular mechanisms of skeletal muscle atrophy are not completely clarified. Therefore, it is difficult to prescribe the right medicine. Moreover, drugs like enzyme inhibitors can interfere with the homeostasis of protein synthesis and there are many uncertainties in their administration. Gene therapy is suitable for muscle atrophy caused by gene mutations with low prevalence but not universal. New genetic drugs need to be further developed. At present, some emerging methods, such as optogenetic technology, biomaterials, and cell therapy, are worthy of continuous exploration. A combination treatment of the above types of therapies may be a better choice. For example, the combination of TFAM and exercise can achieve better results in the treatment of skeletal muscle atrophy. The addition of testosterone adjuvant to movement-based physical rehabilitation therapy can improve musculoskeletal recovery and neuroplasticity in spinal cord injury-induced muscle atrophy, which has better benefits for neuromusculoskeletal recovery than any strategies alone [215]. Skeletal muscle atrophy occurs due to a variety of causes and its molecular mechanism has not yet been fully understood. Therefore, the clinical treatment of muscle atrophy is still a huge challenge. Research on the molecular mechanism of muscle atrophy needs to be further explored to provide new potential targets for the development of new anti-muscle atrophy drugs.

## Figures and Tables

**Table 1:** Drugs in the treatment of skeletal muscle atrophy.

	Drug/ Compound	Mechanism of Action	Function	Ref.
Active Substances of Traditional Chinese Medicine	Magnesium Lithospermate B	Activation of the PI3K-Akt-FoxO1 pathway and inhibition of the TNF-α/TNFRI/NF-κB pathway Inhibition of MAFbx- and MuRF1-mediated muscle degeneration	Alleviates obesity-related myasthenia gravis	[20]
	Puerarin	Activation of AKT/mTOR and inhibition of autophagy	Anti-diabetic rat muscle atrophy	[21,22]
	Tetramethylpyrazine	Inhibition of Ca^2+^/reactive oxygen species increase and subsequent protein hydrolysis and apoptosis	Alleviates waste-induced muscle atrophy	[23]
	mulberry leaf flavone	Increases levels of p-AMPK and PGC-1α; Improvement of insulin resistance and mitochondrial function	Anti-type 2 diabetes mellitus	[24]
	Ginsenosides	Promotes myogenic cell differentiation; Regulates the Akt/mTOR/FoxO3 pathway	Protects dexamethasone -treated C2C12 myotube	[25]
	Triptolide	Up-regulates protein synthesis signals; Downregulates ubiquitin-proteasome, autophagy-lysosome-related molecules and inflammatory mediators	Alleviates LPS-induced inflammation and skeletal muscle atrophy	[26,27]
	Salidroside	Inhibits overproduction of ROS and pro-inflammatory cytokines; Reduce expression of Foxo3A and inhibition of UPS and ALP activation	Alleviates denervation-induced muscle atrophy	[28,29,30]
	Baicalin	Reversal of mitochondrial dysfunction with reduced expression of Cytochrome c and apoptosis-inducing factor; Reversal of caspase-3 and caspase-9 activation	Protect c2c12 myoblast against apoptosis	[31]
	Corylifol A	Activates p38 MAPK pathway; Inhibition of NF-κB-mediated E3 ligase mechanism and activation of Akt	Protects myotubes against dexamethasone damage	[32]
Chemical Drugs	Metformin	Reduce intramuscular lipid sediments, and increase glucose utilization through the AMPK/Sirt1 pathway	Alleviates skeletal muscle atrophy in grx1 ko mice	[33,34]
	Lithium chloride	Inhibition of expression of inflammation-related factors and atrophy genes	Therapeutic effects on sepsis-induced muscle atrophy and cancer cachexia.	[35,36]
	Imidazolo-oxindole	Anti-inflammatory; Attenuates MuRF1 and MAFbx expression; Promotes protein synthesis through AKT/mTOR/S6K1 axis	Therapeutic effects in C2C12 myotubes and LPS-treated mouse skeletal muscle	[37]
Antioxidants	Resveratrol	Reversal of mitochondrial dysfunction via PKA/LKB1/AMPK pathway; Reversal of oxidative stress	Therapeutic effects on sarcopenia	[38]
	Carnosol	Inhibition of TNF-α/NF-κB pathway; anti-malignant effects	Alleviates muscle atrophy and fat lipolysis induced by cancer cachexia	[39]
	Isoquercetin	Reversal of oxidative stress; Conversion of skeletal muscle from slow to fast fiber types	Protective effect on denervated muscle atrophy	[9]
	*N*-acetyl-_L_-cysteine	Reduces ROS levels in mouse muscle Increase myosin heavy chain	Alleviates denervation or fasting-induced skeletal muscle atrophy	[3]
	Pyrroloquinoline quinone	Inhibition of Jak2/STAT3, TGF-β1/Smad3, JNK/p38 MAPK and NF-κB signaling pathways	Alleviates denervation or fasting-induced muscle atrophy	[40]
Hormone Drugs	Testosterone	Stimulates the Ras/MEK/ERK pathway and inhibits MSTN expression; Inactive FoxO	Protects C2C12 skeletal muscle cells against apoptosis	[41,42]
Enzyme Inhibitors and Activators	Aspirin	Regulates STAT3 inflammatory signaling pathway; Regulates the Sirt1/PGC-1α signaling axis	Alleviates denervation-induced muscle atrophy	[43]
	Celecoxib	Inhibition of skeletal muscle inflammation and oxidative stress; Inhibition of ALP and UPS; Improves blood flow	Alleviates denervation-induced muscle atrophy	[44]
	Trichostatin A	Activates SMN2 gene expression Inhibit HDAC4/MYOG/FoxO axis; Downregulates UPS, markers of apoptosis; Partially preventing the reduction of type I and type IIa fibers	Increases survival of SMA mouse; Anti-atrophy induced by cigarette smoke exposure and unloading	[45,46,47,48]
	Rolipram	Decrease MAFbx and MuRF1 levels; Decrease the activity of calpain and caspase-3	Alleviates skeletal muscle atrophy in diabetes mellitus rats	[49]
	Roflumilast	Upregulates of the NRF2, sirtuin-1 pathway; increases cAMP signaling; decrease UPS and MSTN gene expression	Therapeutic effect of muscle wasting in patients with COPD	[50]
	Torbafylline(HWA 448)	Anti-inflammation; Activates PDE4/cAMP/Epac/PI3K/AKT pathway	Alleviates skeletal muscle atrophy caused by injury, denervation, diabetes mellitus, cancer, and sepsis	[51]
	Pentoxifylline	Activates cAMP and AKT; Inhibits the expression of atrogenes and calpain/caspase-3	Mitigates the loss of muscle mass in diabetic rats	[52]
	ALKBH5	Demethylation of HDAC4 mRNA and stabilization of HDAC4 mRNA; Block the activation of FoxO3	Alleviates denervation-induced muscle atroph	[53,54,55]

**Table 2:** Stem Cell and Exosome Therapy for skeletal muscle atrophy.

	Cells/ Exosomes	Mechanism of Action	Limitation	Ref.
Muscle-Derived Stem Cells	Satellite cells	Produces progeny and form muscle fibers for transplantation into the defective area	/	[120,121]
	CD133+ angioblasts	Repopulated ecological niches of satellite cells, regenerative response after injury	Cells are few and fragiles; limited delivery efficience	[122,123]
	Pericytes	Release of nutritional factors; Modulates local immune response	lack optimal harvesting organ and strategies	[124,125]
Non-Muscle-Derived Stem Cells	Bone marrow-derived MSCs (BM-MSCs)	Improve muscle contraction	uncertainties regarding the paracrine effect of MSC, clinical optimization, and CM manufacturing process standards	[126,127]
	Allogeneic placenta-derived MSCs (PL-MSCs)	Reduces fibrosis and inflammation		[128]
	Umbilical cord-derived MSCs (UC-MSCs)	Reduced expression of atrophy-related protein; inhibition of ROS production		[129,130]
	IPSCs	Activation of myogenic signaling; Induces regenerating myogenic progenitor cells	The residual epigenetic memory from the somatic donor cell source may reduce the pluripotency of the generated cell line, leading to a biased differentiation potential	[131,132,133]
Exosomes	Exosomes from satellite cells transduced with Ad-miR29	Downregulation of YY1 and TGF-β pathway proteins	The follow-up time for treatment was relatively short	[134,135]
	UC-MSC-EVs	Release of circHIPK3 serves as a miR-421 sponge to inhibit inflammation, increases FOXO3a expression and prevents the activation of inflammasome	/	[136]
	Exosomes from differentiated human skeletal myoblasts	Regulate skeletal myogenesis through the transfer of diverse myogenic factors; Reduces the fibrotic area and increased the number of regenerated myofibers	Unknown about the key factors in controlling cell fate and promoting skeletal muscle regeneration; The adverse effects may arise from high doses of exosomes (e.g., cell apoptosis)	[137,138]
	SKP-SC-EVs	Reduces ROS production and inflammation; Downregulate UPS and ALP; Improves microcirulation	/	[6]
	exosomes from human BM-MSCs	Inhibit dexamethasone-induced muscle atrophy via miR486-5p/Foxo1 Axis	/	[139]
